# Detection of SARS-CoV-2 in schools using built environment testing in Ottawa, Canada: A multi-facility prospective surveillance study

**DOI:** 10.1371/journal.pone.0300397

**Published:** 2024-05-17

**Authors:** Nisha Thampi, Tasha Burhunduli, Jamie Strain, Ashley Raudanskis, Jason A. Moggridge, Aaron Hinz, Evgueni Doukhanine, Lucas Castellani, Rees Kassen, Janine McCready, Caroline Nott, Alex Wong, Michael Fralick, Derek R. MacFadden

**Affiliations:** 1 Department of Pediatrics, University of Ottawa, Ottawa, Ontario, Canada; 2 CHEO Research Institute, Ottawa, Ontario, Canada; 3 Lunenfeld-Tanenbaum Research Institute, Sinai Health System, Toronto, Ontario, Canada; 4 Department of Biology, University of Ottawa, Ottawa, Ontario, Canada; 5 Department of Biology, Carleton University, Ottawa, Ontario, Canada; 6 DNA Genotek Incorporated, Ottawa, ON, Canada; 7 Sault Area Hospital, Sault Ste. Marie, Ontario, Canada; 8 Clinical Sciences Division, Northern Ontario School of Medicine, Sudbury, Ontario, Canada; 9 Michael Garron Hospital, Toronto East Health Network, Toronto, Ontario, Canada; 10 The Ottawa Hospital Research Institute, Ottawa, Ontario, Canada; 11 Department of Medicine, University of Ottawa, Ottawa, Ontario, Canada; 12 Institute for Advancing Health Through Agriculture, Texas A&M AgriLife, Fort Worth, Texas, United States of America; 13 Division of General Internal Medicine, Sinai Health System, Toronto, Ontario, Canada; Sidra Medical and Research Center, QATAR

## Abstract

Classroom and staffroom floor swabs across six elementary schools in Ottawa, Canada were tested for SARS-CoV-2. Environmental test positivity did not correlate with student grade groups, school-level absenteeism, pediatric COVID-19-related hospitalizations, or community SARS-CoV-2 wastewater levels. Schools in neighbourhoods with historically elevated COVID-19 burden showed a negative but non-significant association with lower swab positivity.

## Background

Environmental surveillance for SARS-CoV-2 has been an effective public health tool for monitoring COVID-19 in the population, with the most studied approach being wastewater sampling [1]. Surface sampling within the built environment may be a more spatially refined approach for surveillance in specific contexts. For example, a recent multi-centre study across 10 long-term care homes demonstrated that the percentage of positive swabs for SARS-CoV-2 correlates with cases and outbreaks of COVID-19 [2]. A strong correlation between environmental sampling and cases of COVID-19 has also been shown in hospital settings [3, 4]. While SARS-CoV-2 has been detected in non-healthcare settings such as schools, it is unclear whether trends correlate with community prevalence [5–7]. We sought to determine the relationship between SARS-CoV-2 swab positivity in the school environment and community indicators of COVID-19 prevalence.

## Methods

A prospective surveillance study was conducted in a convenience sample of six publicly funded elementary schools over a 12-week period, from March 28 to June 17, 2022, in Ottawa, Ontario. Floors of every classroom, gymnasium and staff lounge in each school were sampled twice weekly by research or school staff trained by the research team, with a minimum two-day interval between most collection times. Sites identified by the research team and school administration were in high-traffic areas, including entrances, vestibules, and handwashing stations in the room. The same 2-by-2-inch areas of the floors were swabbed each time and at the same time of day (late morning or early afternoon), with one sample collected from each room and processed by the laboratory within the week of collection using previously validated protocols [2, 3]. Schools were cleaned at the end of the day. Wastewater data were obtained from publicly available sources; weekly means and 95% confidence intervals (CIs) were calculated from daily N1 and N2 target values, with the quantity of SARS-CoV-2 RNA expressed as a proportion relative to Pepper mild mottle virus RNA [8]. Permission was received from boards and principals prior to each school visit, and school communities were notified by the principal prior to starting sample collection.

COVID-19 vaccine eligibility had been extended to children ages 5 to 11 years in November 2021. Ontario lifted mandatory masking policies in schools and other indoor settings apart from public transit on March 21, 2022, when students were set to return to in-person learning following Spring Break. School-based mask recommendations shifted from mandatory to strongly recommended. The largest Ottawa school board reintroduced mandatory masking between April 13 and May 30 in response to high community prevalence of SARS-CoV-2. In Ontario, students enroll in Grade 1 in the year of their sixth birthday. Thus, the youngest child in Junior Kindergarten may be 3 years old, and the oldest child in Grade 8 may be 14 years old.

The main outcome was SARS-CoV-2 detection on floor surfaces. Data were analyzed at the level of the student classroom, staffrooms and school, and compared to publicly available data on historical SARS-CoV-2 burden in Ottawa neighbourhoods served by these schools, as well as school-level student and staff absenteeism rates, city-level wastewater signals, and number of COVID-19-associated pediatric hospitalizations at CHEO, the sole pediatric acute care facility in the region, which was submitted daily to the Ontario Ministry of Health [8–10]. During this period, testing was restricted to children admitted with infectious symptoms. Ethics review was waived by the research ethics board of the University of Ottawa as no identifiable human data were collected. Written informed consent for visiting the school and performing floor sampling was received from the school administration prior to the initial site visit.

Statistical analyses were performed using the R programming language (v4.2.2). Confidence intervals on proportions (positivity, absenteeism rates) were computed using the Wilson method. Generalized linear mixed models were created using ‘glmer’ from the R package ‘lme4’ (v1.1–29). Classroom-level data were grouped into Junior and Senior Kindergarten, Grades 1–3, Grades 4–6, and Grades 7–8 for analysis of age group effects. Swab positivity and absenteeism rates were grouped into semi-weekly bins to compute school-level means for correlation analysis. We calculated the Spearman correlation coefficient (*r*) between the presence of SARS-CoV-2 on floors (as a positive/negative result and as weekly geometric mean of viral copies), absenteeism, pediatric hospitalizations, and levels of SARS-CoV-2 detected in community wastewater surveillance.

## Results

**Fig 1:**
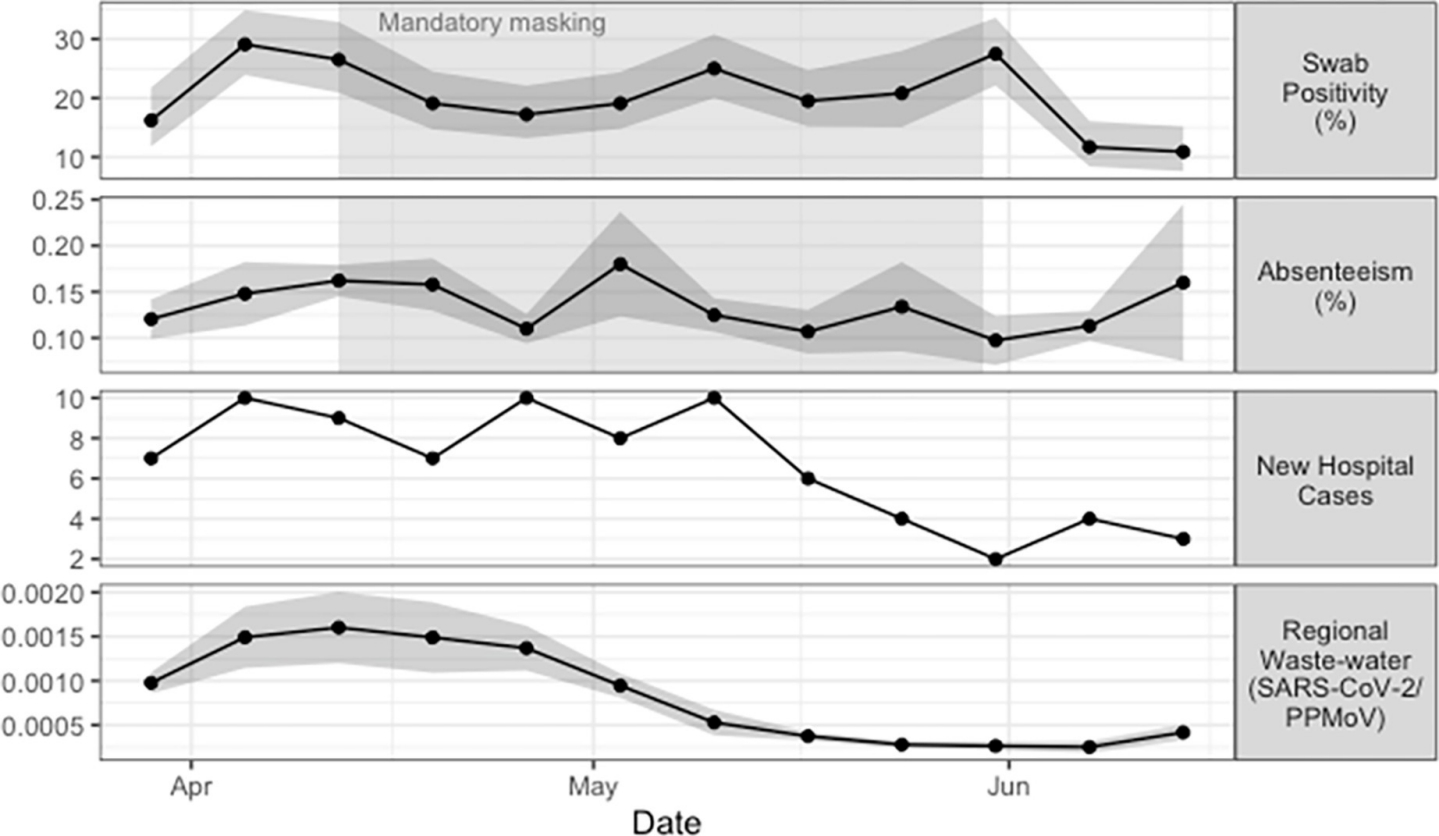
Weekly time-series (panels top to bottom): weekly mean positivity of environmental PCR tests from swabs collected at six schools (% and 95% CI); weekly mean absenteeism at the same schools (% and 95% CI); weekly counts of new SARS-CoV-2 cases at the regional children’s hospital; and Ottawa regional waste-water SARS-CoV-2 detection relative to PPMoV (mean and 95% CI of N1 and N2 targets). Points display either means or count values, shaded ribbons around the means represent the 95% CI, where applicable. Shaded boxes indicate the period during which mandatory masking policies were in effect at four of the six schools.

During the 12-week study period, 2,860 floor samples were collected across six schools. Overall test positivity for SARS-CoV-2 RNA was 20%, with rates ranging from 10% to 36% among schools (median 16.5%) and from 0% to 77.8% among rooms (median 15.8%). Weekly aggregated floor-swab positivity across schools remained between 10–30% over the study period, with small peaks observed during early-to-mid-April, early-to-mid-May, and early June (Fig 1). Four schools had mandatory masking policies in place between April 13 and May 30, 2022. We found no correlation between swab positivity and absenteeism rates within each school, irrespective of the presence of mandatory masking policies (S1 Fig), nor were there any sampling sites that were consecutively positive for SARS-CoV-2 at the classroom level beyond one to two weeks.

There was no significant correlation between the weekly floor-swab positivity of schools and regional wastewater signal, school absenteeism rates, or number of pediatric COVID-19-related hospitalizations during the same week (Spearman’s *r* = 0.235, -0.039, and 0.285 respectively; *p* > 0.05; Fig 1). Similarly, absenteeism rates were not significantly correlated with wastewater signal or hospitalizations (Spearman’s *r* = 0.52 and 0.25, respectively; *p* > 0.05); however, the regional wastewater detection and pediatric COVID-19-related hospitalizations were strongly correlated during the study period (Spearman’s *r* = 0.75; *p* = 0.005). Though not significant, there was a trend towards overall floor-swab positivity rates at the school level being negatively correlated with the prevalence of COVID-19 prior to 2022 in the surrounding neighbourhood (Spearman’s *r* = -0.6, *p* = 0.24; Table 1). Floor swab positivity did not change significantly by day of the week when floors were swabbed (S1 Table in S1 File). There was no significant correlation between viral copies and absenteeism (Spearman’s r = -0.063, p = 0.85) nor between viral copies and COVID-19-related hospitalizations (Spearman’s r = 0.336, p = 0.29).

**Table 1:** Summary of surface surveillance of SARS-CoV-2 conducted at six schools and historical case rates for the surrounding neighbourhoods.

Site	Earliest	Latest	Collection Days	N	Positives	Historical Covid-19 Burden (per 100,000)^1^
School 1	2022-03-28	2022-06-16	17	238	32 (13.4%)	5,699
School 2	2022-04-05	2022-06-16	18	233	101 (43.3%)	2,887
School 3	2022-03-29	2022-06-16	23	617	59 (9.6%)	5,547
School 4	2022-04-01	2022-06-17	21	587	92 (15.7%)	2,831
School 5	2022-04-01	2022-06-17	19	266	46 (17.3%)	3,682
School 6	2022-03-29	2022-06-16	23	919	242 (26.3%)	3,064

^1^ Data from Ottawa Neighbourhood Study in neighbourhoods from July 1, 2020 to December 31, 2021 [10].

A sensitivity analysis using multivariable Poisson regression of variables to predict weekly new pediatric cases is shown in S2 Table in S1 File and aligns with the findings from correlation analysis.

Swab positivity was also examined at the grade level by classroom (S2 Fig). Staff rooms, followed by classrooms with children in Junior and Senior Kindergarten generally had higher swab positivity, while Grades 7–8 classrooms had lower swab positivity throughout the period of study compared to other classrooms, however there was no significant difference by age group (S3 Table in S1 File). A likelihood ratio test of nested mixed models failed to find a significant fixed effect of age (*p* = 0.28), suggesting that apparent differences in swab positivity rates between age groups arise from the differences in the composition of age group (*e*.*g*., roughly 50% of the grades 7–8 classrooms are situated in School #3, which has the lowest positivity among schools).

## Discussion

In our 12-week prospective study across six publicly funded elementary schools, SARS-CoV-2 was detected 20% of the time on floors of classrooms, gyms, and staff areas, and did not correlate with student and staff absenteeism or community wastewater signals at the school or aggregate level. A negative but non-significant correlation was found between test positivity in schools and prior COVID-19 infection rates in neighbourhoods which may have served as proxy for community immunity among student populations. The variability in test positivity among schools suggest hyper-localization of SARS-CoV-2 transmission during this period.

Strengths of the study include serial sampling of floors during a community surge of SARS-CoV-2, examination of the impact of school-based mandatory masking policies, and comparisons with community metrics, namely wastewater signals, historical neighbourhood COVID-19 infection rates, and pediatric COVID-19 disease requiring hospitalization.

School-based surveillance of respiratory infections contributes to understanding the burden and dynamics of infection transmission through schools and workplaces, households and social networks [6, 11]. Environmental surveillance has been proposed as an additional layer to inform pandemic and endemic disease surveillance, given the limitations of human-based testing as a surveillance strategy to inform public health action [1].

Wastewater testing has demonstrated usefulness in assessing SARS-CoV-2 levels in the community and acute care, however it can lack spatial resolution [1]. In built environments, reliable detection of SARS-CoV-2 has been shown with sampling of floors compared to other surfaces [3, 7]. In long-term care homes, serial floor sampling may be particularly relevant as not all residents use toilets, and detection on floors was shown to predate COVID-19 outbreaks by days and even weeks [2].

There have been numerous studies examining environmental presence of SARS-CoV-2 in school settings, however viral detection was infrequent, with test positivity ranging from 0% to 5%, despite the presence of student cases identified by clinical and asymptomatic screen testing [5–7]. As there was limited access to molecular testing in the community and lack of public reporting of cases, we examined publicly-reported absenteeism rates as a proxy measure for burden of illness among students and staff. However absenteeism reporting is highly variable across jurisdictions, with differences in ascertainment, reporting, and thresholds to trigger public health action [12, 13].

While environmental sampling of floors has been shown to correlate with COVID-19-related clinical activity in hospital and LTC settings, we did not find a correlation between viral copies and absenteeism or pediatric hospitalizations, at the grade- or school-level, and did not test for correlation between rooms within each school. Also, PCR-based detection cannot discriminate among fresh or relic RNA, or RNA brought into buildings via fomites, and may not be useful for surveillance of active viral shedding at a single point in time [7, 14, 15]. Notably our outcomes were not true measures of COVID-19 burden within individual schools, and there may be value for establishing trends and following progression in test positivity as a reflection of community pediatric burden. There was a lack of correlation among sites and age groups that may reflect immunity through vaccination and/or prior exposure to SARS-CoV-2. Finally, the study was not adequately powered to estimate effect of mandatory masking. We were unable to identify any significant correlation between environmental surveillance testing for SARS-CoV-2 in schools and several measures of COVID-19 activity. The role of surveillance in the built environment warrants further study to determine its usefulness in informing public health action to reduce the burden of communicable diseases.

## Supporting information

S1 Checklist STROBE statement—checklist of items that should be included in reports of observational studies. (DOCX)

S1 Fig Time-series of biweekly aggregated absenteeism and floor swab positivity rates at the school level. Shaded areas indicate the period in which mandatory masking policies were reinstituted at the school level.
(TIF)

S2 Fig Weekly swab positivity aggregated by grade. Shaded areas represent 95% confidence intervals.
(TIF)

S1 File (DOCX)
