# Supplementary material for: Detection of SARS-CoV-2 in schools using built environment testing in Ottawa, Canada: A multi-facility prospective surveillance study
Source: PLoS One. 2024 May 17;19(5):e0300397. doi: 10.1371/journal.pone.0300397 (PMC11101119; doi:10.1371/journal.pone.0300397)
Supplement: S1 Checklist — (DOCX) [file pone.0300397.s001.docx]

STROBE Statement—checklist of items that should be included in reports of observational studies

|  | Item No. | Recommendation | Page  No. | Relevant text from manuscript |
| --- | --- | --- | --- | --- |
| **Title and abstract** | 1 | (*a*) Indicate the study’s design with a commonly used term in the title or the abstract | 1 | Multi-Facility Prospective Surveillance Study |
|  |  | (*b*) Provide in the abstract an informative and balanced summary of what was done and what was found | 2 | Classroom and staffroom floor swabs across six elementary schools in Ottawa, Canada were tested for SARS-CoV-2. Schools in neighbourhoods with historically elevated COVID-19 burden had lower environmental swab positivity. Environmental test positivity did not correlate with student grade groups, school-level absenteeism, pediatric COVID-19-related hospitalizations, or community SARS-CoV-2 wastewater levels. |
| Introduction | | | |  |
| Background/rationale | 2 | Explain the scientific background and rationale for the investigation being reported | 3 | Surface sampling within the built environment may be a more spatially refined approach for surveillance in specific contexts.  While SARS-CoV-2 has been detected in non-healthcare settings such as schools, it is unclear whether trends correlate with community prevalence. |
| Objectives | 3 | State specific objectives, including any prespecified hypotheses | 4 | We sought to determine the relationship between SARS-CoV-2 swab positivity in the school environment and community indicators of COVID-19 prevalence. |
| Methods | | | |  |
| Study design | 4 | Present key elements of study design early in the paper | 4 | A prospective surveillance study was conducted in six publicly funded elementary schools over a 12-week period |
| Setting | 5 | Describe the setting, locations, and relevant dates, including periods of recruitment, exposure, follow-up, and data collection | 4 | Six publicly-funded elementary schools  March 28 to June 17, 2022  Floors of every classroom, gymnasium and staff lounge in each school were sampled twice weekly by research or school staff trained by the research team, with a minimum two-day interval between most collection times. |
| Participants | 6 | (*a*) *Cohort study*—Give the eligibility criteria, and the sources and methods of selection of participants. Describe methods of follow-up  *Case-control study*—Give the eligibility criteria, and the sources and methods of case ascertainment and control selection. Give the rationale for the choice of cases and controls  *Cross-sectional study*—Give the eligibility criteria, and the sources and methods of selection of participants | 4-5 | Publicly-funded schools  Wastewater data from publicly available sources  Permission was received from boards and principals prior to each school visit, and school communities were notified by the principal prior to starting sample collection. |
|  |  | (*b*) *Cohort study*—For matched studies, give matching criteria and number of exposed and unexposed  *Case-control study*—For matched studies, give matching criteria and the number of controls per case |  |  |
| Variables | 7 | Clearly define all outcomes, exposures, predictors, potential confounders, and effect modifiers. Give diagnostic criteria, if applicable | 5 | The main outcome was SARS-CoV-2 detection on floor surfaces. |
| Data sources/ measurement | 8* | For each variable of interest, give sources of data and details of methods of assessment (measurement). Describe comparability of assessment methods if there is more than one group | 4-5 | References 8, 9, 10 |
| Bias | 9 | Describe any efforts to address potential sources of bias | 5  6 | Data were analyzed at the level of the student classroom, staffrooms and school.  Classroom-level data were grouped into Junior and Senior Kindergarten, Grades 1-3, Grades 4-6, and Grades 7-8 for analysis of age group effects. |
| Study size | 10 | Explain how the study size was arrived at | 4 | study was conducted in a convenience sample of six publicly funded elementary schools |

Continued on next page

| Quantitative variables | 11 | Explain how quantitative variables were handled in the analyses. If applicable, describe which groupings were chosen and why | 5-6 | Data were analyzed at the level of the student classroom, staffrooms and school, and compared to publicly available data on historical SARS-CoV-2 burden in Ottawa neighbourhoods served by these schools, as well as school-level student and staff absenteeism rates, city-level wastewater signals, and number of COVID-19-associated pediatric hospitalizations at CHEO, the sole pediatric acute care facility in the region, which was submitted daily to the Ontario Ministry of Health  Classroom-level data were grouped into Junior and Senior Kindergarten, Grades 1-3, Grades 4-6, and Grades 7-8 for analysis of age group effects.  Swab positivity and absenteeism rates were grouped into semi-weekly bins. |
| --- | --- | --- | --- | --- |
| Statistical methods | 12 | (*a*) Describe all statistical methods, including those used to control for confounding | 5-6 | Statistical analyses were performed using the R programming language (v4.2.2). Confidence intervals on proportions (positivity, absenteeism rates) were computed using the Wilson method. Generalized linear mixed models were created using ‘glmer’ from the R package ‘lme4’ (v1.1-29).  We calculated the Spearman correlation coefficient (r) between the presence of SARS-CoV-2 on floors, absenteeism, pediatric hospitalizations, and levels of SARS-CoV-2 detected in community wastewater surveillance. |
|  |  | (*b*) Describe any methods used to examine subgroups and interactions | 6 | Swab positivity and absenteeism rates were grouped into semi-weekly bins to compute school-level means for correlation analysis. We calculated the Spearman correlation coefficient (r) between the presence of SARS-CoV-2 on floors, absenteeism, pediatric hospitalizations, and levels of SARS-CoV-2 detected in community wastewater surveillance. |
|  |  | (*c*) Explain how missing data were addressed |  | There were no missing data with twice weekly sampling strategy among the six schools |
|  |  | (*d*) *Cohort study*—If applicable, explain how loss to follow-up was addressed  *Case-control study*—If applicable, explain how matching of cases and controls was addressed  *Cross-sectional study*—If applicable, describe analytical methods taking account of sampling strategy |  | n/a |
|  |  | (*e*) Describe any sensitivity analyses |  | none |
| Results | | | | |
| Participants | 13* | (a) Report numbers of individuals at each stage of study—eg numbers potentially eligible, examined for eligibility, confirmed eligible, included in the study, completing follow-up, and analysed | 4 | Participants were six publicly-funded elementary schools in Ottawa |
|  |  | (b) Give reasons for non-participation at each stage |  | n/a – schools were chosen by convenience sampling |
|  |  | (c) Consider use of a flow diagram |  |  |
| Descriptive data | 14* | (a) Give characteristics of study participants (eg demographic, clinical, social) and information on exposures and potential confounders | 6 | Weekly aggregated floor-swab positivity across schools remained between 10-30% over the study period, with small peaks observed during early-to-mid-April, early-to-mid-May, and early June.  Four schools had mandatory masking policies in place between April 13 and May 30, 2022. |
|  |  | (b) Indicate number of participants with missing data for each variable of interest |  |  |
|  |  | (c) *Cohort study*—Summarise follow-up time (eg, average and total amount) | 6 | 12-week study period, 2,860 floor samples were collected across six schools. |
| Outcome data | 15* | *Cohort study*—Report numbers of outcome events or summary measures over time | 6 | Overall test positivity for SARS-CoV-2 RNA was 20%, with rates ranging from 10% to 36% among schools (median 16.5%) and from 0% to 77.8% among rooms (median 15.8%). |
|  |  | *Case-control study—*Report numbers in each exposure category, or summary measures of exposure |  |  |
|  |  | *Cross-sectional study—*Report numbers of outcome events or summary measures |  |  |
| Main results | 16 | (*a*) Give unadjusted estimates and, if applicable, confounder-adjusted estimates and their precision (eg, 95% confidence interval). Make clear which confounders were adjusted for and why they were included | 6 | There was no significant correlation between the weekly floor-swab positivity of schools and regional wastewater signal, school absenteeism rates, or number of pediatric COVID-19-related hospitalizations during the same week (Spearman’s r = 0.235, -0.039, and 0.285 respectively; p > 0.05; fig. 1). Similarly, absenteeism rates were not significantly correlated with wastewater signal or hospitalizations (Spearman’s r = 0.52 and 0.25, respectively; p > 0.05); however, the regional wastewater detection and pediatric COVID-19-related hospitalizations were strongly correlated during the study period (Spearman’s r = 0.75; p = 0.005). Overall floor-swab positivity rates at the school level were not correlated with the prevalence of COVID-19 prior to 2022 in the surrounding neighbourhood (Spearman’s r = -0.6, p = 0.24; Table 1). |
|  |  | (*b*) Report category boundaries when continuous variables were categorized |  |  |
|  |  | (*c*) If relevant, consider translating estimates of relative risk into absolute risk for a meaningful time period |  |  |

Continued on next page

| Other analyses | 17 | Report other analyses done—eg analyses of subgroups and interactions, and sensitivity analyses | 7 | Swab positivity was also examined at the grade level by classroom (Supplemental Figure 2). Staff rooms, followed by classrooms with children in Junior and Senior Kindergarten generally had higher swab positivity, while Grades 7-8 classrooms had lower swab positivity throughout the period of study compared to other classrooms. A likelihood ratio test of nested mixed models failed to find a significant fixed effect of age (p = 0.28), suggesting that apparent differences in swab positivity rates between age groups arise from the differences in the composition of age group (e.g., roughly 50% of the grades 7-8 classrooms are situated in School #3, which has the lowest positivity among schools). |
| --- | --- | --- | --- | --- |
| Discussion | | | | |
| Key results | 18 | Summarise key results with reference to study objectives | 7 | In our 12-week prospective study across six publicly funded elementary schools, SARS-CoV-2 was detected 20% of the time on floors of classrooms, gyms, and staff areas, and did not correlate with student and staff absenteeism or community wastewater signals at the school or aggregate level. |
| Limitations | 19 | Discuss limitations of the study, taking into account sources of potential bias or imprecision. Discuss both direction and magnitude of any potential bias | 8-9 | we did not find a correlation at the grade- or school-level. Also, PCR-based detection cannot discriminate among fresh or relic RNA, or RNA brought into buildings via fomites, and may not be useful for surveillance of active viral shedding at a single point in time.(7,14,15) |
| Interpretation | 20 | Give a cautious overall interpretation of results considering objectives, limitations, multiplicity of analyses, results from similar studies, and other relevant evidence |  | Notably our outcomes were not true measures of COVID-19 burden within individual schools, and there may be value for establishing trends and following progression in test positivity as a reflection of community pediatric burden. |
| Generalisability | 21 | Discuss the generalisability (external validity) of the study results | 9 | The role of surveillance in the built environment warrants further study to determine its usefulness in informing public health action to reduce the burden of communicable diseases. |
| Other information | |  | | |
| Funding | 22 | Give the source of funding and the role of the funders for the present study and, if applicable, for the original study on which the present article is based |  | This work was supported by funding from a Canadian Institutes of Health Research Operating Grant [EGA 179419]. |

*Give information separately for cases and controls in case-control studies and, if applicable, for exposed and unexposed groups in cohort and cross-sectional studies.

**Note:** An Explanation and Elaboration article discusses each checklist item and gives methodological background and published examples of transparent reporting. The STROBE checklist is best used in conjunction with this article (freely available on the Web sites of PLoS Medicine at http://www.plosmedicine.org/, Annals of Internal Medicine at http://www.annals.org/, and Epidemiology at http://www.epidem.com/). Information on the STROBE Initiative is available at www.strobe-statement.org.
