# Supplementary figures and images for: Detection of SARS-CoV-2 in schools using built environment testing in Ottawa, Canada: A multi-facility prospective surveillance study
Source: PLoS One. 2024 May 17;19(5):e0300397. doi: 10.1371/journal.pone.0300397 (PMC11101119; doi:10.1371/journal.pone.0300397)

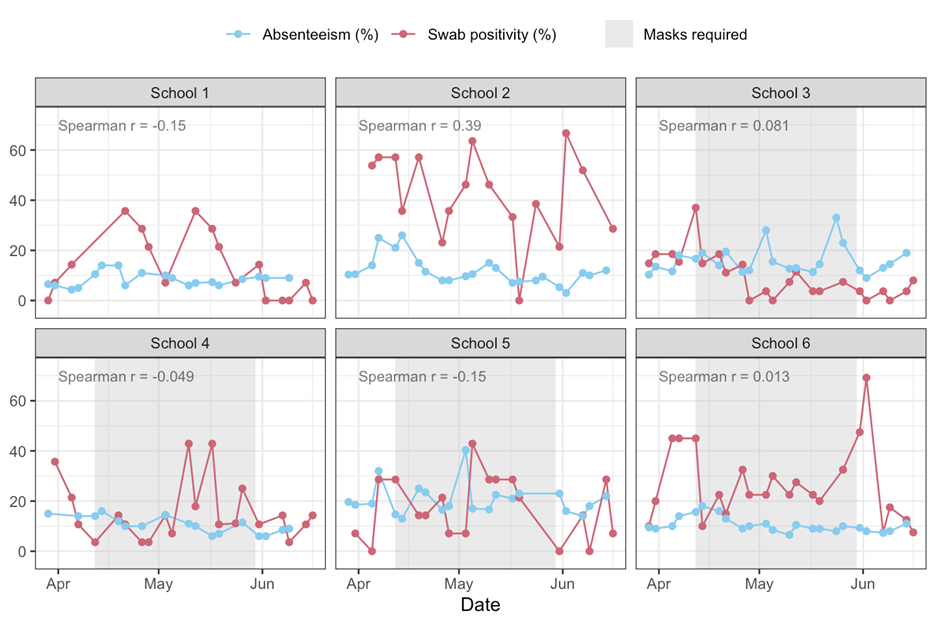

Supplement: S1 Fig — Shaded areas indicate the period in which mandatory masking policies were reinstituted at the school level. (TIF) [file pone.0300397.s002.tif]

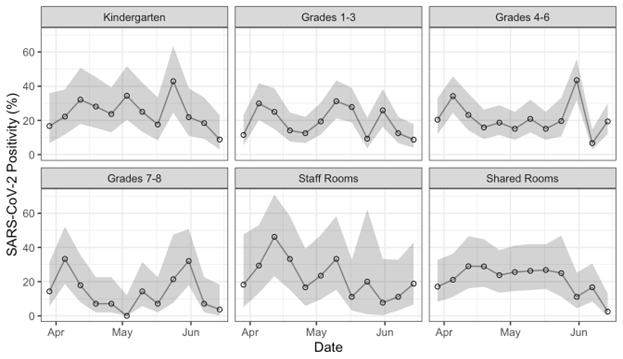

Supplement: S2 Fig — Shaded areas represent 95% confidence intervals. (TIF) [file pone.0300397.s003.tif]
