# Supplementary material for: Detection of SARS-CoV-2 in schools using built environment testing in Ottawa, Canada: A multi-facility prospective surveillance study
Source: PLoS One. 2024 May 17;19(5):e0300397. doi: 10.1371/journal.pone.0300397 (PMC11101119; doi:10.1371/journal.pone.0300397)
Supplement: S1 File — (DOCX) [file pone.0300397.s004.docx]

**Detection of SARS-CoV-2 in Schools Using Built Environment Testing in Ottawa, Canada: A Multi-Facility Prospective Surveillance Study**

Nisha Thampi, Tasha Burhunduli, Jamie Strain, Ashley Raudanskis, Jason A. Moggridge, Aaron Hinz, Evgueni Doukhanine, Lucas Castellani, Rees Kassen, Janine McCready, Caroline Nott, Alex Wong, Michael Fralick, Derek R. MacFadden

**Supplemental Information File**

S1 Table. Swab positivity by day of week

S2 Table. Incidence rate ratios for predictors of weekly new cases

S3 Table: Floor swab positivity by age group.

S1 Fig: Time-series of biweekly aggregated absenteeism and floor swab positivity rates at the school level.

S2 Fig: Weekly swab positivity aggregated by grade.

**S1 Table. Swab positivity by day of week**

| **Characteristic** | | **Odds ratio** | **95% confidence interval** | **p-value** |
| --- | --- | --- | --- | --- |
| Day of the week | |  |  |  |
|  | Friday | - | - |  |
|  | Wednesday | 1.09 | 0.77, 1.54 | 0.6 |
|  | Tuesday | 1.34 | 0.76, 2.36 | 0.3 |
|  | Thursday | 1.15 | 0.66, 2.02 | 0.6 |
|  | Monday | 0.91 | 0.39, 2.13 | 0.8 |

**S2 Table. Incidence rate ratios for predictors of weekly new cases**

| **Characteristic** | **Incidence Rate Ratio** | **95% Confidence Interval** | **p-value** |
| --- | --- | --- | --- |
| Wastewater | 1.79 | 1.07, 2.98 | 0.025 |
| Floor swab positivity | 1.01 | 0.97, 1.05 | 0.6 |
| Absenteeism | 0.98 | 0.89, 1.09 | 0.7 |

**S3 Table: Floor swab positivity by age group.**

| **Characteristic** | | **Odds ratio** | **95% confidence interval** | **p-value** |
| --- | --- | --- | --- | --- |
| Age group | |  |  |  |
|  | Grades 4-6 | - | - |  |
|  | Grades 1-3 | 0.86 | 0.59, 1.26 | 0.4 |
|  | Grades 7-8 | 0.67 | 0.39, 1.16 | 0.2 |
|  | Kindergarten | 1.01 | 0.64, 1.58 | >0.9 |
|  | Shared rooms | 1.35 | 0.86, 2.12 | 0.2 |
|  | Staff rooms | 1.14 | 0.61, 2.13 | 0.7 |

**S1 Fig: Time-series of biweekly aggregated absenteeism and floor swab positivity rates at the school level**. Shaded areas indicate the period in which mandatory masking policies were reinstituted at the school level.

**S2 Fig: Weekly swab positivity aggregated by grade.** Shaded areas represent 95% confidence intervals.
